# The role of add-on strategies (cognitive enhancers, cognitive remediation)

**DOI:** 10.1192/j.eurpsy.2025.212

**Published:** 2025-08-26

**Authors:** G. Sachs, A. Erfurth

**Affiliations:** 1 Medical University of Vienna; 2 1st Department of Psychiatry and Psychotherapeutic Medicine, Klinik Hietzing, Vienna, Austria

## Abstract

**Abstract:**

Cognitive impairments are present from the first manifestations of the disorder, including social cognition in individuals at ultra-high risk for psychosis (UHR). While advances in psychopharmacology in recent decades have significantly altered the course of the illness, antipsychotic therapy is more effective in treating positive symptoms than negative symptoms, comorbid depression, and cognitive and social cognitive dysfunction. Again, cognitive impairment has a significant impact on functional outcome, more so than symptom severity.

Due to the particular significance of cognitive deficits, it is very important to identify them accurately at an early stage. Strategies for identifying cognitive deficits are presented in an EPA guidance paper.

To improve cognition in patients with schizophrenia, it has been suggested to combine pharmacotherapy with neuropsychological training. A meta-analysis showed the positive effect of cognitive remediation programmes in schizophrenia. While the strategies available today for the treatment of cognitive dysfunction are useful and should be implemented, it is hoped that pharmacological add-on strategies will come onto the market to have a greater effect on cognitive impairments.

**Disclosure of Interest:**

None Declared

